# COVID-19 certificate as a cutting-edge issue in changing the perception of restaurants’ visitors—Illustrations from Serbian urban centers

**DOI:** 10.3389/fpsyg.2022.914484

**Published:** 2022-10-05

**Authors:** Tamara Gajić, Marko D. Petrović, Ivana Blešić, Dragan Vukolić, Ilija Milovanović, Milan Radovanović, Darko B. Vuković, Marija Kostić, Nikola Vuksanović, Slavica Malinović Milićević

**Affiliations:** ^1^Geographical Institute “Jovan Cvijić” SASA, Belgrade, Serbia; ^2^Institute of Sports, Tourism and Service, South Ural State University, Chelyabinsk, Russia; ^3^Faculty of Hotel Management and Tourism, University of Kragujevac, Vrnjačka Banja, Serbia; ^4^Department of Geography, Tourism and Hotel Management, Faculty of Sciences, University of Novi Sad, Novi Sad, Serbia; ^5^Department of Psychology, Faculty of Philosophy, University of Novi Sad, Novi Sad, Serbia; ^6^Department of Economics and Industrial Engineering, Perm National Research Polytechnic University, Perm, Russia; ^7^Department of Hospitality, Higher Education School for Management and Business Communication, Novi Sad, Serbia

**Keywords:** COVID-19, BFF, restaurants, consumer, behavior, Serbia

## Abstract

As one of the first European cases of the introduction of COVID-19 certificates, the Serbian Government initiated the measure of limited working hours of restaurants for unvaccinated visitors. Due to such actions and frequent bans on working during the pandemic, many restaurants in Serbia had to lay off workers or close. At the end of October 2021, the certificate for entering restaurants and all catering facilities for all the visitors became mandatory. It is interesting to note that earlier findings suggested that some personality characteristics determine the specific behaviors during the pandemic, but there is still a small number of results related to restaurants’ visitors. This study aimed to investigate the predictive strength of the Big Five Factors (BFF) to attitudes toward visits to restaurants in Serbia during the pandemic, depending on the attitudes toward accepting COVID-19 certificates. A survey was conducted on a total sample of 953 visitors of restaurants in three major cities in Serbia. The results of hierarchical regression analysis showed that Openness and Extraversion positively predict attitudes toward visits to facilities during a pandemic, while Conscientiousness and Neuroticism were negative predictors. However, in the second step of hierarchical regression analysis, attitudes toward a COVID-19 certificate as a mediator variable significantly reduced the negative effect of Neuroticism on the attitudes toward visits. It seems that, by obtaining the certificate, the fear of unsafe stays in restaurants can be reduced, and that making decisions about (no) visiting restaurants during the pandemic does not necessarily have to be compromised by emotional lability.

## Introduction

Due to the global pandemic of COVID-19, and the closing of borders, tourism and catering suffered great damage during 2019 until today. National and local authorities have assessed various sanctions on their citizens, involving restraining orders, travel bans, stay-at-home orders, self-isolation, and other daily limitations, and more recently COVID-19 passes for all facilities and movements (Gössling et al., 2021; Podra et al., 2021; Xiang et al., 2021; Aleksić et al., 2022). Epidemiologists in Serbia have identified cafes and restaurants as the riskiest places for the spread and transmission of the coronavirus (Gajić et al., 2021a,b). In the period of October 2021, the idea came from the authorities and the crisis medical staff, to introduce COVID-19 certificates and thus ensure the operation of restaurants and cafes in safer conditions (Gajić et al., 2020). The problem arose due to numerous theories and ignorance, whether the vaccinated transmit the pandemic, and whether it makes sense to introduce such permits to enter catering facilities. Many restaurants were closed at exactly 8 pm, so as not to differentiate between vaccinated and unvaccinated visitors. In accordance with numerous studies of the impact of the pandemic on the development of tourism and services, the authors conducted a study of visits to restaurants in several cities in Serbia during the pandemic, using the BFF (OCEAN) model of personality traits. The study aimed to identify personality profiles that better predict visits to tourism and hospitality facilities during COVID-19, with or without passes.

The global hospitality sector has been hit hard by the crisis brought about by COVID-19. It can be seen that the economic sector of consumption has suffered damage, and there have been major changes in consumer behavior, which is essential for the recovery of the hospitality sector (Gajić et al., 2020). The hospitality industry took the hardest hit, resulting in the current closure of many restaurants or adaptation to new concepts to make it easier for consumers to return as easily as possible after the pandemic (Gursoy and Chi, 2020; Nepal, 2020; Oliveira et al., 2020; Sigala, 2020; Božović et al., 2021). It is most necessary to determine the behavior of consumers in risky situations, and based on this, to determine measures for better and smooth operation of the restaurant (Toubes et al., 2021). Some of the measures for recovery after emergencies can be implemented through increased flexibility and creativity, investing in a communication platform, and selling gift cards (Warerkar, 2020; Norris et al., 2021), as well as through certain consumer preferences such as special rooms in restaurants (Kim and Lee, 2020). There are studies where the results indicate that a pandemic negatively moderate the effect of cultural experience and introduce novelty on the intention to consume (Dedeoğlu et al., 2022).

A study conducted by Hakim et al. (2021) points out that consumers’ propensity to visit restaurants during a pandemic is influenced by risk perception and different levels of trust. According to the results, the component with the greatest effect is trust in restaurants and brands. In addition, denial of the disease has a positive effect on restaurant visits during the pandemic.

The Big Five model (OCEAN) has been extensively discussed in management research and consumer behavior in specific situations, monitoring of job performance, especially when it comes to unforeseen and crisis situations (Lauriola and Levin, 2001; Saihani et al., 2009; Power and Pluess, 2015; Chien, 2019; Blešić et al., 2021). Each of the basic dimensions includes a number of specific personality traits and preferences for certain patterns of behavior: neuroticism, extraversion, openness, agreeableness, and conscientiousness (Black, 2000; John et al., 2008). The openness trait describes people who have a wide range of interests, curiosity, aspirations for independence, and are readier to take risks (Jamrozy et al., 1996; Card et al., 2003). Extraversion is characterized by irritability, sociability, talkativeness, assertiveness, and a large amount of emotional expressiveness (Rice, 1997; Copley, 2004; Cobb-Clark and Schurer, 2012; Khalil, 2016), with confirmed theories that greater extroversion is associated with lower rational style of decision—making (El Othman et al., 2020). Conscientiousness could predict job performance of frontline employees of restaurants (Tracey et al., 2007), because they will better manage multiple requests (Teng, 2008; Young and Corsun, 2009). In consumer behavior, agreeableness largely predicts self-esteem when making important decisions, where they very easily exchange information with each other and thus form their decision (Oh, 1997; Litvin et al., 2008; Deniz, 2011). Neuroticism is a trait characterized by sadness, low mood and emotional instability, with a high tendency for high neuroticism scores to contribute to lower risk in decision making (Lauriola and Levin, 2001; McCrae et al., 2005; Tanasescu et al., 2013).

A huge number of studies link the Big Five model with consumer purchasing behavior and leisure activities, which include choice of a brand, trips to restaurants, and tourist trips (Horton, 1979; Church and Katigbak, 2002; Barnett, 2006; Barnett and Klitzing, 2006; McCrae, 2010). People with a higher degree of agreeableness and conscientiousness, had negative direct effects on the perception of readiness for health risk and acceptance of health—risky behaviors (Vollrath et al., 1999). Research has confirmed that people with pronounced extraversion and conscientiousness traits, make important decisions easier and faster, both at work and in certain specific situations (Erjavec et al., 2019), while those with openness choose special services (Kvasova, 2015; Verma, 2017).

In many businesses, owners need to be able to understand the profile and desires of their customers, in order to tailor their business to meet customer needs during crises, especially in tourism and hospitality sector (Wee and Chia, 2012; Tran et al., 2015; Köşker et al., 2018; Chien, 2019). Knowledge of personality traits has a marketing role in creating products for consumers in tourism and hospitality, but they are also important for determining job satisfaction among hotel and restaurant workers (Young and Corsun, 2009; Jani, 2014; Yildirima et al., 2016). The dimensions of extraversion and openness have been observed to be weak predictors, between personality traits and job satisfaction among hotel workers (Yildirima et al., 2016). It is very important to study the behavior of potential visitors for the selection of services. Openness, extroversion and neuroticism can predict the psychographic positions of tourist destinations (Amet, 2017).

When looking at the relationship between personality types and going to destinations during a pandemic, there is recent research that claims that neuroticism and conscientiousness negatively influenced travel intentions, but are positively correlated with extroversion and openness (Tepavčević et al., 2021). Greater openness, conscientiousness and neuroticism showed the most consistent associations when talking about destinations during a pandemic, and pointed out that the pattern of connections between personality traits and precautionary behaviors varied depending on specific behavior (Airaksinen et al., 2021). The same results revealed that personality explains only a small fraction (between 0.6 and 3.8%) of variance in the four outcomes, perception of infection risk, behavior change to prevent infection, belief in the effectiveness of policies to combat further coronavirus spread and trust relevant policy makers and institutions. Agreeableness and its aspect of trust have shown the strongest associations (Rammstedt et al., 2021). However, it must be pointed out that there is a lack of literature on research on people’s attitudes toward vaccination that uses OCEAN theory. Only a few studies have investigated the impact of personality traits on their attitudes toward vaccination (Guidry et al., 2021; Shmueli, 2021; Suess et al., 2022), but nothing concrete can be concluded about their intention to visit a restaurant, and whether that visit implied the obligation of having a certificate. The largest percentage of respondents, more than two—thirds of Americans, believe in the health benefits of vaccination, while about one—tenth of respondents are concerned about its safety (Lin and Wang, 2020). The results obtained in the study by Halstead et al. (2020), indicate that in five personality types, there are large differences in the perception of the importance of vaccination. For neuroticism they received different results, from completely positive to completely negative answers, while for some personality traits they did not get the expected results at all. The use of vaccines from different manufacturers that were on the market (BioNTech, Sinovac, Moderna, Sputnik V) is considered to have created a sense of security in people, and they are trying to free themselves and adapt to new living conditions or so-called new normalcy (Contreras, 2020). In this context of the new way of life, the attitude of tourists is implied that they want to go on vacation even though the infection still reigns and despite the risk of COVID-19 infection. Also, some theorists claim that the decisions and intentions of tourists to visit restaurants are influenced by a specific combination of fear of infection and the desire to travel and visit restaurants (Dedeoğlu et al., 2022). So, the assumption is that the personality traits of the guests will influence their perception of a safe visit to a restaurant, which we observe through the prism of immunization (Blešić et al., 2021). Seçilmiş et al. (2021) in their research on the impact of consumer confidence in vaccines, claim that there are no significant results which confirm that trust in vaccines creates a strong decision to visit a tourist destination or a restaurant. They further argue that conscientiousness and openness are significantly positively correlated with attitudes toward the health benefits of vaccination. Attitudes toward services and consumers of tourist and hotel services vary depending on the type of personality (Tekin and Kalkan, 2017). One major critique of the theory is highlighted by MCrae (2010), who argues that the existence of dynamic processes is quite prominent in the theory but not explained in detail. Some research indicates that the intention of consumers, in the COVD-19 situation, to visit restaurants is influenced by: fear of disease, trust in health surveillance, marketing, consumer perception and demographic characteristics (Hakim et al., 2021). A large part of the respondents were in favor of introducing certificates for travel purposes, but they were against mandatory certificates in other work activities (Drury et al., 2021).

Given the importance of personality traits in making decisions and specific behavior in crisis situations, based on the above literature, the main goal of this study was to investigate how certain psychological groups of people perceive the risk during their stay in restaurants in the period of a pandemic. The starting hypothesis is that personality traits significantly describe the percentage of positive attitudes toward restaurants during a pandemic. Moreover, the study examines whether a COVID-19 certificate has a role in reducing fear in making decisions about visiting restaurants, which is another hypothesis of the research. Also, one of the important assumptions in the research is that vaccination could be a significant mediator when it comes to certain psychological groups, such as neuroticism. The assumption is that in some groups the vaccine would have an effect on reducing fear, while in some groups it would have absolutely no effect.

## Materials and methods

### Sample and procedure

The authors of the manuscript, together with 50 scientific associates—students of tourism and catering and psychology, conducted a trial pilot study, in the period from October to December 2021. They distributed questionnaires through online surveys, to the respondents from three cities in Serbia: Belgrade, Novi Sad, Niš. The required sample size was calculated using G*power (Faul et al., 2009). Taking into account that there was a total of 6 predictors (5 independent in the first step, and 1 inserted in the second step of the applied analysis) and 1 criterion, the required effect size was set to η^2^ = 0.15, with a statistical power of 0.95, and it was calculated that a sample size of 472 respondents may be appropriate for this research. However, out of the distributed number of questionnaires, which was 3,000, we received answers from 953 respondents, although for the representativeness of the sample, the number of respondents should have been much higher due to number of citizens from three town which were included in this research. The sample was voluntary, i.e., we had no other criteria for collecting respondents, due to pandemic, so we collected a certain number of people from all three cities. We could not examine people live, because of the pandemic, since we did not want to put anyone at risk of infection, neither the members of our team, nor the people who participated in the research. The distribution of respondents is as follows: 350 from Belgrade, 314 from Novi Sad, and 289 from Niš. According to the results of the chi-square test (x^2^ = 5.774, *p* = 0.0566), there is no statistically significant difference in the representation of respondents from the three urban centers. Of the total sample, 45.8% were male and 54.2% were female. There were 31.8% of the participants in 18–25 age range, 38.7% in 26–30 age range, 14.9% in 36–50 age range, and 14.6% of participants were more than 50 years old. The study was anonymous and no confidential details that could identify the respondents were gathered. All the items were exemplified on a 5-point Likert scale by answering from 1 = absolutely disagree to 5 = completely agree.

## Measures

### Personality traits

The authors used the BFF measurement model (OCEAN questionnaire) with 22 statements, proposed by John and Srivastava (1999). OCEAN questionnaire is aimed to measure Big Five personality traits: Extraversion (5 items; α = 0.76), Neuroticism (3 items; α = 0.74), Openness (4 items; α = 0.64), Conscientiousness (5 items; α = 0.78), and Agreeableness (5 items; α = 0.73).

### Attitudes toward COVID-19 vaccination certificates

These attitudes were measured through the assessment of participants’ answers to the situation of usability and cost of COVID-19 certificates. Questions for assessing these attitudes in this research were: “The rule with having a COVID-19 certificate is very useful,” “Everyone should have a COVID-19 certificate, so that we can return to normal life as soon as possible,” “People should not be allowed into enclosed spaces if they do not have a COVID-19 certificate,” “COVID-19 certificates will contribute to a faster end of the pandemic,” and “I think that COVID-19 certificates are the best measure to fight against the pandemic.” These questions form a unique score of positive attitudes toward COVID-19 certificates (α = 0.68).

### Visiting restaurants during the pandemic

Questions for assessing these attitudes in this research were: “The pandemic doesn’t stop me from visiting restaurants,” “My daily life is accompanied by going to restaurants, even during a pandemic,” “Most people should visit restaurants during a pandemic,” “I can’t imagine going to a restaurant at least once a week even though it’s a pandemic,” and “A pandemic should not prevent restaurants from having the typical flow of people on a daily basis.” Those 5 questions form a unique score of positive attitudes toward visiting restaurants during COVID-19 pandemic (α = 0.76).

### Data analysis

The calculations were completed in IBM SPSS 21 statistical software. With the aim to test the mediating role of attitudes toward COVID-19 vaccination certificates between personality traits and attitudes toward visiting hospitality facilities during the COVID-19 pandemic, we conducted a hierarchical multiple regression analysis.

The predictors in the first step of the analysis were personality traits, while in the second step we included attitudes toward COVID-19 vaccination certificates. All effect sizes were interpreted according to Cohen’s report (1988). Hierarchical regression was chosen because it is the best way to show if our predictor variables (personality traits) explain a statistically significant amount of variance in attitudes toward visiting restaurants after accounting for attitudes toward COVID-19 vaccination certificates. Although we could use multiple dependent variables SEM analysis, which allows variables to correlate, regression analysis adjusts for other variables in the model, which is in line with our aim to examine the partial, individual effects of personality traits, controlling the remaining traits. Besides that, descriptive statistics and correlation analysis were performed in order to get an insight of the distribution and relationship between the variables. We decided to conduct hierarchical regression, since the SEM model could not converge toward a solution in relation to the collected data. The collected data are completely anonymized and are available on the OSF repository, and can be obtained at the request of the authors. Since all data (dependent and independent variables) were collected using the same method, there were potentially the risk of common method bias (CMB). Using the Harman’s single factor test, we got the result that all the items explain 7.45% of single-factor solution, so CMB was not a pervasive issue for this research due to small amount of shared variance among the variables due to the method (Fuller et al., 2016).

## Results

### Descriptive statistics and correlation analysis

Basic descriptive statistics parameters for all measurements are presented in Table 1, as well as the correlation between measures and gender differences. The frequency distribution of numerical features was examined with skewness and kurtosis indicators. Given that all the variables are normally distributed, parametric statistics methods were used. According to Tabachnick and Fidell (2013), all the variables were normally distributed (Sk and Ku are in range −1.5 to 1.5). Significant gender differences in favor of women were shown. There were no gender differences on measures of attitudes toward visits and certificates.

**TABLE 1:** Descriptive statistics, gender differences and intercorrelation of variables.

	O	C	E	A	N	AtC	AtV	Males	Females
O	−							3.00	3.01
C	0.09**	−						3.52	3.50
E	−0.10**	−0.13**	−					4.28	4.23
A	−0.09**	−0.17**	0.08*	−				3.36	3.44*
N	−0.13**	−0.17**	0.12**	0.02	−			3.88	4.04**
AtC	0.04	0.01	0.01	0.04	−0.07*	−		3.50	3.57
AtV	0.09**	–0.06	0.08*	0.02	0.06	0.11**	−	2.98	2.94
*M*	3.00	3.51	4.25	3.40	3.97	3.54	2.96		
*SD*	1.02	0.75	0.76	0.62	0.78	0.62	0.54		
*Sk*	0.01	–0.05	–1.17	0.20	–0.68	0.00	0.09		
*Ku*	–1.06	–0.41	0.88	–0.23	–0.20	–0.45	0.04		

O, Openness; C, Conscientiousness; E, Extraversion; A, Agreeableness; N, Neuroticism; AtC, Attitudes toward COVID-19 vaccination certificates; AtV, Attitudes toward visiting hospitality facilities during COVID-19 pandemic; M, mean; SD, standard deviation; Sk, skewnees; Ku, kurtosis.

*p < 0.05. **p < 0.01.

Attitudes toward visiting restaurants during COVID-19 pandemic made positive, but modest, correlations with Openness and Extraversion, and also with attitudes toward COVID-19 vaccination certificates. On the other hand, attitudes toward COVID-19 vaccination certificates made a negative and modest correlation only with Neuroticism. It seems that people with higher Openness and Extraversion are actually more inclined to have more positive attitudes toward visiting restaurants during the COVID-19 pandemic, i.e., that the needs for socialization, sensations, and new experiences are actually potential predictors of approachable behavior in the context of visiting restaurants despite the global pandemic. On the other hand, it seems that people who are less inclined to intensely experience unpleasant emotions such as fear, sadness, etc., are also more inclined to see the positive sides of vaccination as a protective factor in the fight of hospitality sector against the pandemic. When interpreting correlations, it must be borne in mind that, due to a potential problem with sample representativeness, relationships between variables may suffer from the effect of overinflation.

### Hierarchical multiple regression analysis

Results suggested that personality traits could explain a significant, although small percentage (2%) of the attitudes toward visiting restaurants during the COVID-19 pandemic. Openness and Extraversion predict attitudes toward visiting restaurants during the COVID-19 pandemic in a positive way, while Neuroticism and Conscientiousness have negative relations with those attitudes. After the inclusion of attitudes toward COVID-19 vaccination certificates in Step 2, personality traits could still explain a significant percentage (7%) of the attitudes toward visiting hospitality facilities during the COVID-19 pandemic, but the role of Neuroticism declines, which means that the positive attitudes toward the COVID-19 vaccination certificates represent a protective factor for people with high Neuroticism who have expressed a need for visiting restaurants during the pandemic. Therefore, it seems possible to form a specific profile of people who have a positive attitude toward visiting restaurants during the pandemic, which consists of traits such as a tendency to socialize, experiencing sensations, openness to new experiences, and less conscientious forms of behavior, but at the same time who are prone to protective behaviors like getting a vaccine that inhibits unpleasant emotional responses like sadness, fear, etc. in the context of the COVID-19 pandemic. However, bearing in mind that the sample did not, in a representative manner, include residents of these three cities, these findings should be taken with caution and interpreted partially as a random effect that may not represent a fully adequate reflection of the real situation. This is partially indicated by the low degree of explanation of the criterion variance by the included predictors in both steps.

## Discussion

Depending on the psychological group of respondents, motivation may be increased or decreased by the impact of a vaccination certificate for visiting a restaurant. This study tried to hypothesize the answer to how certain psychological groups perceive the attitude toward visiting restaurants during the pandemic, in relation to having a vaccination certificate. A modified Big Five personality traits model was used (John and Srivastava, 1999). There are few studies that show that vaccines can reduce fear in people and encourage them to return to normal life. The topic of vaccination is on the very margins of social communication or a topic on which no one speaks freely. However, some research shows that human fear and mental instability caused by the pandemic will have no impact on further life and that everything will be normalized very quickly. Also, some research claims that fear of a pandemic does not influence decisions about visiting restaurants. Seçilmiş et al. (2021) found no evidence that trust in the vaccination moderates the association between cognitive response with trust, and the intention to visit a destination. Bremser et al. (2021), proved that, despite the potential for excitement and confusion associated with this contagious disease, people were willing to travel during periodic restrictions on entry and exclusion of travel and that the benefits of such travel outweighed barriers such as wearing masks, social distancing, and other restrictive measures. With this pilot study, the authors tried to examine the relationship of psychological groups to the decision to visit restaurants during the pandemic, depending on the importance of the certificate. The obtained results can be rather interpreted as initial assumptions (due to small sample size, compared to the population of the country) that personality traits, which were investigated according to the Big Five model, explain a significant percentage of attitudes toward visiting restaurants during the pandemic. It is a preliminary pilot study, where a total of 3,000 surveys were distributed in three cities of Serbia, and a total of 953 responses were received. The sample taken is entirely voluntary, as it is a pilot study. Another way of collecting the sample would be risky for the groups of respondents, but also for the researchers themselves, due to the possibility of infection with the corona virus during that period, and because of the restrictions that existed. For the statistical model of data processing, the authors chose Hierarchical Regression, because this model can best see the strength of the predictor variable (personality traits) in explaining statistically significant variance in attitudes toward restaurant visits. Attitudes toward restaurant visits during the pandemic were shown to have positive but modest correlations with Openness and Extraversion, as well as attitudes toward COVID-19 vaccination certificates. However, attitudes toward COVID-19 vaccination certificates had a negative and modest correlation only with Neuroticism. Table 2 provides an insight into the results of hierarchical regression analysis, where the results show that five personality traits according to the Big Five personality traits (OCEAN) theory could explain a small, but significant percentage of attitudes toward restaurant visits during the COVID-19 pandemic. In the following paragraphs, the results and confirmations of the hypotheses from the given tables will be described in a little more detail, for each type of person separately, with reference to their character traits.

**TABLE 2:** Hierarchical multiple regression analysis for attitudes toward visiting hospitality facilities during COVID-19 pandemic.

Variables	Attitudes toward visiting hospitality during COVID-19 pandemic			
	β	95% CI		*P*
		Lower	Upper	
**Step 1**				
O	0.10	0.02	0.09	0.00
C	–0.07	–0.10	–0.01	0.04
E	0.09	0.02	0.11	0.01
A	0.01	–0.04	0.07	0.67
N	–0.07	–0.09	–0.01	0.04
*R* = 0.15; Adjusted *R*^2^ = 0.02**; Δ*R*^2^ = 0.02**				
**Step 2**				
O	0.10	0.02	0.08	0.00
C	–0.07	–0.10	–0.01	0.04
E	0.08	0.01	0.10	0.01
A	0.01	–0.05	0.06	0.77
N	–0.06	–0.09	–0.01	0.08
AtC	0.11	0.03	0.14	0.00
*R* = 0.28; Adjusted *R*^2^ = 0.07**; Δ *R*^2^ = 0.05**				

O, Openness; C, Conscientiousness; E, Extraversion; A, Agreeableness; N, Neuroticism; AtC, Attitudes toward COVID-19 vaccination certificates; β, standardized partial contruibution of predictor; R, multiple correlation coefficient; R^2^, multiple determination coefficient; ΔR^2^, change of multiple determination coefficient after AtC introduction into model; CI, confidence interval.

**p < 0.01.

The Openness feature showed statistical significance in predicting restaurant visits during the pandemic. People who are high in Openness tend to have many of the following characteristics: creative, intelligent and knowledgeable, give great attention to mental imagery, interested in new things, enjoy hearing new ideas, like thinking about abstract concepts, usually more liberal and open to diversity, interested in artistic endeavors, adventurous (Weisberg et al., 2011). They may also be more willing to engage in risky behaviors, including visits to destinations and restaurants during crisis situations (Sutin et al., 2013). However, it is necessary to keep in mind that a convenient, voluntary sample of respondents participated in this research, and it is not possible to assume with certainty whether the same results would have been obtained if the sample included people who do not use the Internet, who are less open to new technologies and who, perhaps due to computer illiteracy or computer anxiety, did not dare to participate in this research.

Statistical significance was also observed in the predictor of Conscientiousness, but its correlation was in the negative direction. It turns out that the more conscientious people are, the less they will visit restaurants during the pandemic. When someone is conscientious, they are able to apply self-discipline and self-control in order to follow and eventually achieve their goals. Conscientious people may become too serious and may need a gentle push to cheer up and have fun (Denissen et al., 2018). They can also burn out by overwork, become overly rigid or inflexible, and struggle to be spontaneous. If someone scores high in conscientiousness, they are likely to carefully consider all the facts before making decisions. They tend to think through most things and consider the consequences before finally acting (Martin et al., 2007), which can be linked to behavior during the pandemic. Nevertheless, bearing in mind the characteristics of a potentially unrepresentative sample, it is possible that these effects can be compared to random ones, considering that most of the research involves highly conscientious people, who have the need to contribute to scientific results through their engagement. It is not excluded that the representativeness of the sample may have been compromised by the fact that the sample does not include those people who are less conscientious about their obligations, are less informed about current events such as the pandemic, and are less inclined to order and discipline.

When looking at the predictor of Extraversion, there is statistical significance in predicting restaurant visits during a pandemic, in a positive direction. It means that the more extraverted visitors are, the more will they visit restaurants regardless of the pandemic. People who have a high level of extroversion need social stimulation to feel full of energy (Mccabe and Fleeson, 2012). They simply gain energy by engaging in social interaction. Extraversion significantly contributes to the anticipation of going to restaurants without a certificate. Additionally, bearing in mind that the sample may include those people who are more open to social engagement and the need for stimulation, as well as that there are fewer older people in the sample who have a less pronounced need for openness to socializing due to aging, these findings should interpret with caution.

Agreeableness is a person’s ability to put other people’s needs above their own and struggling in situations that require difficult decisions. They can also be slow in judging other people and often care about people unconditionally. The agreeableness predictor does not show statistical significance in predicting restaurant visits, and even after the introduction of the COVID-19 certificate, the significance does not change.

Neuroticism is a negative personality trait that includes maladaptation and negative emotions, poor self-regulation, or the ability to manage instincts, problems coping with stress, a strong reaction to perceived threats, and a propensity to complain (Costa and McCrae, 1999). For the predictor of Neuroticism, statistical significance is at the very limit of acceptable value. With the introduction of mediators, the role of neuroticism is declining, which means that a positive attitude toward vaccination gives strength to people with high neuroticism when visiting restaurants during a pandemic. Neuroticism is also associated with such precautions in general in many studies during the pandemic period (e.g., Airaksinen et al., 2021). The results showed that Neuroticism is related to current mental health and decisions during a pandemic. It is concluded vaccines can play a great role in making decisions in people with high Neuroticism (Shokrkon and Nicoladis, 2021). Considering that the sample is potentially unrepresentative, because it does not include, for example, computer-anxious people in whom neuroticism is higher, or it does not include people who have a pronounced fear of viruses, which can be one of the manifestations of neuroticism, these findings should rather be taken with caution in the context of a pilot study, rather than as final conclusions regarding this personality trait.

## Limitations

There were some limitations during the research. Some of the limitations are procedural and analytical, and others are theoretical. Considering the type of analytical research, one of the key procedural limitations is the inability to go out on the field and survey restaurant visitors. Questionnaires were distributed online, because it was the safest way of research and, in general, a way of communicating with people, due to the prevailing a pandemic, and of course to reduce the risk of infection, both for the respondents and the researchers. The sample used in the research (953) is entirely voluntary, due to the limited way of communication with the respondents. In this way, an almost equal number of questionnaires was obtained from all the three cities (although the cities are significantly different in size and number of inhabitants), which allows us to assume and give freedom of choice to this number of samples as a type of representative sample. The research is pilot in type, and for that reason, a smaller number of respondents was used than it should be according to the total population from these cities. A limiting circumstance is the access to respondents (a certain number of people are of an older age, and do not have the necessary knowledge and skills to use social networks and computers). We believe that this sample is sufficient for a pilot study. Field research reduces the number of missing values, and increases the number of valid answers. Online research takes more time, gives low response rates, as many ignore the emails and surveys they receive. In the field research, the possibility to get all the answers is greater, because it depends on the personal contact of the interviewers and respondents. Many potential participants in the research who belong to old age group are reluctant to participate precisely because of their poor knowledge of technology, lack of access to online services, inaccessibility of computers, and a version to technology. Analytic the limitation is also the fact that we do not have data on who of the respondents was vaccinated and who was not, and we do not have data on whether they went to some visits to restaurants during the pandemic, and how often did it. In this context, we could not segment respondents into vaccinated and unvaccinated. It is assumed that there are limitations in terms of obtaining socially desirable answers in the research. The topic or question of vaccination is something that few would be free to comment on. Both sides are subject to condemnation, both vaccinated and unvaccinated. Therefore, getting a valid and honest answer, especially in written online form, is almost impossible. So, we can consider all this as a pilot study, i.e., some initial step of considering the profile of people who are inclined to take risks or not take risks in pandemic situations in relation to their dispositional factors, i.e., in relation to personality traits. Also, it can be argued that there are limitations in theoretical terms, as there is not much research on specific issues. Very few studies have dealt with the topic of vaccination during COVID-19, and the influence of personality theory on the assessment of their perception of the vaccine, and whether the vaccine affects their determination to visit restaurants and travel. It can be said that there are almost no such studies, which with this model, the theory of personality, investigates the position on the impact of vaccination certificates on decision-making and intentions. Future research should relate to the wider area of research, not only in Serbia, and they should relate to other factors, not only the ones that influence the intention to visit restaurants.

## Conclusion

The COVID-19 pandemic has caused great damage to the global industry, including tourism and hospitality industry. It is considered to be a global health, social, and economic emergency (Fonseca et al., 2021). Tourism and hospitality industry suffered a major decline during the pandemic of the last 2 years in the world and in Serbia. Restrictions and social distancing policies have had a dramatic effect on the industry. Tourism completely collapsed at some point, and the hospitality industry was at risk, maintaining itself barely enough not to close facilities completely. Many restaurants and cafes have been forced to limit working hours, close facilities due to unprofitability and high costs, and distribute layoffs to employees. At a time when the idea of COVID-19 certificates was developed, there seemed to be some salvation for the operation of catering facilities. It is believed that the restaurant sector will recover in the next 3 years. Motivation is just one of the factors that helps in making decisions (Abbacete et al., 2012). Previous studies point to the fact that when people see high risk, trust has a stronger effect on creating intentions in their behavior (Anderson and Karunamoorthy, 2003). According to a recent study, travelers are more inclined to avoid travel during the pandemic owing to perceived health risks (Zheng et al., 2021).

As the virus spread across the countries, there was a huge distribution of new information. Anxiety and agony have reigned among rumors and true knowledge (Torales et al., 2020). Guidry et al. (2021) consider that the attitude toward the vaccine is subjective and that people decide to take it if they could receive some benefits from the vaccine and obtaining a certificate. However, no research uses OCEAN theory to investigate the impact of vaccine certification on the decision to visit restaurants and similar facilities. The model was used in research to determine whether some personality types would want to get vaccinated or not, but the model was not used to determine the impact of vaccines and certificates on restaurant intentions (Shmueli, 2021). In their study, Suess et al. (2022), give results on how much tourists are willing to be vaccinated so that they can travel, but not through the Big Five model. They proved that the model of health beliefs defines the future activity of tourists. It is assumed that similar results would be obtained when visiting restaurants. The obtained results assume that personality traits explain a good percentage of attitudes toward visiting restaurants during a crisis or pandemic. Openness and Extraversion positively predict the attitude toward restaurant visits during pandemic, while Neuroticism and Conscientiousness have a negative attitude toward restaurant visits. After the introduction of the mediator variable, the importance of neuroticism decreases, which means that having a vaccination certificate is a protective factor for people with high neuroticism. However, it is necessary to keep in mind that this is a plot study and that the observed effects are potentially random, which may call into question the validity of the conclusions, but at the same time it provides a suitable basis for further research in the context of this topic. The pilot research can contribute to the field of economics and the health sector. The study assumes that having a vaccination certificate affects the intention of visitors to go to restaurants during a pandemic. On the other hand, the perception of risk and some kind of trust in the certificate also affects the intention of consumers.

Research can contribute to the development of personality psychology, in terms of starting assumptions for more significant research. It is possible that such a pilot study will contribute to the understanding of the needs of restaurant visitors during the pandemic. Based on the understanding of their needs and expectations, it would be possible to spot the main problems, and on that basis determine some business measures to avoid situations like losing visitors and closing restaurants. In this way, restaurants will enable the time and principle of work during the crisis, but also the possibility of avoiding conflicts with spheres of interest and government organizations. So far, there are great differences in attitudes in society, which lead to discrimination against people of different understandings. The study contributes to and enhances the existing research and new results, providing empirical knowledge on the impact of risk on restaurant visits. This pilot study can complete to the lack of literature on consumer behavior in crisis situations and pandemic periods, and science will benefit validly from this and similar research in studying the behavior of certain personality types. Therefore, based on these assumptions, future research can focus precisely on specific groups of visitors. Also, many restaurants can direct their business more toward certain groups of visitors. The study can also contribute to the practical implications, as it highlights the way restaurants will operate in the future. Of course, every restaurant will have to change the way it does business and adapt it to unforeseen situations, including the possible return of the pandemic. The way of doing business means finding solutions for non-contact business, online management, as well as improving the system of security measures and protection in restaurants. Knowledge of the existence of risks and the way consumers behave will dictate the future business of restaurants in the world. Thus, the business will be under controlled conditions, ready to take all security measures and reduce the level of risk to a minimum, but also to maintain business during crisis situations. It will create a feeling for visitors and their needs, which are changing in relation to the development of the pandemic. Therefore, they will not lose customers and there will be no closure of facilities. However, research on this topic will prove the practical implications in the segment of destination and business development management, because based on the information obtained from the research, it will deal more with market segmentation and visitor needs. Some of the changed ways of doing business, which were created during the pandemic, will remain in use even after the situation calms down. An example of this is online business and maintaining contact with visitors.

## Data availability statement

The raw data supporting the conclusions of this article will be made available by the authors, without undue reservation.

## Author contributions

TG, IB, MP, and IM contributed to the conception and design of the study and contributed to statistical analysis and data processing. MR and DBV wrote the first draft of the manuscript. DV, MK, and NV planned and coordinated. All authors contributed to manuscript revision, read, and approved the submitted version.
